# Machine Learning Revealed Ferroptosis Features and a Novel Ferroptosis-Based Classification for Diagnosis in Acute Myocardial Infarction

**DOI:** 10.3389/fgene.2022.813438

**Published:** 2022-01-25

**Authors:** Dan Huang, Shiya Zheng, Zhuyuan Liu, Kongbo Zhu, Hong Zhi, Genshan Ma

**Affiliations:** ^1^ Department of Cardiology, Zhongda Hospital, Southeast University, Nanjing, China; ^2^ Department of Oncology, Zhongda Hospital, Southeast University, Nanjing, China

**Keywords:** machine learning, ferroptosis, acute myocardial infarction, early diagnosis, prediction model

## Abstract

Acute myocardial infarction (AMI) is a leading cause of death and disability worldwide. Early diagnosis of AMI and interventional treatment can significantly reduce myocardial damage. However, owing to limitations in sensitivity and specificity, existing myocardial markers are not efficient for early identification of AMI. Transcriptome-wide association studies (TWASs) have shown excellent performance in identifying significant gene–trait associations and several cardiovascular diseases (CVDs). Furthermore, ferroptosis is a major driver of ischaemic injury in the heart. However, its specific regulatory mechanisms remain unclear. In this study, we screened three Gene Expression Omnibus (GEO) datasets of peripheral blood samples to assess the efficiency of ferroptosis-related genes (FRGs) for early diagnosis of AMI. To the best of our knowledge, for the first time, TWAS and mRNA expression data were integrated in this study to identify 11 FRGs specifically expressed in the peripheral blood of patients with AMI. Subsequently, using multiple machine learning algorithms, an optimal prediction model for AMI was constructed, which demonstrated satisfactory diagnostic efficiency in the training cohort (area under the curve (AUC) = 0.794) and two external validation cohorts (AUC = 0.745 and 0.711). Our study suggests that FRGs are involved in the progression of AMI, thus providing a new direction for early diagnosis, and offers potential molecular targets for optimal treatment of AMI.

## Introduction

Acute myocardial infarction (AMI), a myocardial damage event caused by the rupture of atherosclerotic plaque, is a leading cause of death and disability worldwide (Murray et al., 2015; Mozaffarian et al., 2016). Early diagnosis of AMI and interventional treatment can significantly reduce myocardial damage, improve prognosis and reduce mortality (Braunwald, 2012). Although the evaluation of existing myocardial markers is one of the gold-standard techniques for diagnosing AMI, these markers cannot accurately identify patients with AMI owing to limitations in sensitivity and specificity, resulting in a missed opportunity for optimal treatment (Braunwald, 2012). Therefore, it is necessary to identify novel biomarkers for early diagnosis of AMI, thus reducing mortality and improving prognosis.

Increasing evidence suggests that genetic factors play an important role in the progression of AMI (O'Donnell and Nabel, 2011; Kessler et al., 2013). To date, genome-wide association studies (GWASs) have identified a large number of susceptibility loci of AMI. However, the results of GWASs fail to reveal the relative risk of AMI, and only a small proportion of locus alterations can explain the pathogenesis and progression of AMI (Deloukas et al., 2013; Nikpay et al., 2015). Transcriptome-wide association studies (TWASs) can be used to integrate GWAS data with gene expression data to identify significant gene–trait associations (Gusev et al., 2016) and have demonstrated excellent performance in identifying cardiovascular diseases (CVDs) based on CARDIoGRAMplusC4D consortium’s GWAS data on CVDs (Deloukas et al., 2013; Thériault et al., 2018; Zhang et al., 2019).

Ferroptosis is an iron-dependent programmed cell death characterised by the excessive accumulation of lipid hydroperoxide, culminating in overwhelming lipid peroxidation and eventually leading to death (Xie et al., 2016; Stockwell et al., 2017). Numerous studies have reported that induction of ferroptosis in cancer cells has emerged as a promising alternative to tumour therapy, especially in malignancies that are resistant to conventional treatment (Hassannia et al., 2019; Liang et al., 2019). Furthermore, recent studies have suggested that ferroptosis is a major driver of ischaemic injury in the heart (Gao et al., 2015; Stockwell et al., 2017). However, the specific regulatory mechanisms of ferroptosis in the cardiovascular system remain unclear and require further investigation.

In this study, the results of TWAS and messenger RNA (mRNA) expression profiles of patients with AMI were integrated to identify feature genes expressed in peripheral blood samples. Subsequently, ferroptosis-related genes (FRGs) were identified by comparing the obtained FRG expression data. Finally, a robust prediction model for identifying patients with AMI was constructed using multiple machine learning algorithms and validated in two independent AMI cohorts, thus providing new ideas and tools for early diagnosis of AMI.

## Results

### Identification of Feature Genes in the Peripheral Blood of Patients With AMI Using TWAS

After comparing the peripheral blood data from Genotype-Tissue Expression (GTEx) with large-scale GWAS data from CARDIoGRAMplusC4D for CVDs using TWAS, we identified 1,079 feature genes in the peripheral blood of patients with CVD (TWAS, *p* < 0.05). The top 20 identified AMI-related genes in peripheral blood are listed in Table 1, and detailed results are provided in Supplementary Table S1.

**TABLE 1:** The top 20 candidate genes identified by TWAS for AMI.

ID	Chromosome	BEST.GWAS.ID	BEST.GWAS.Z	TWAS.Z	TWAS.P	Comparative tissue
PSRC1	1	rs7528419	−8.028	−6.442171	1.18E-10	Whole Blood
CARF	2	rs6722332	7.38	−6.33E+00	2.41E-10	Whole Blood
RP11-378J18.8	1	rs17163358	−6.711	−5.852904	4.83E-09	Whole Blood
GGCX	2	rs1561198	6.06	5.73E+00	1.01E-08	Whole Blood
POC1B	12	rs2681472	5.81	−5.71972	1.07E-08	Whole Blood
IL6R	1	rs4845618	5.284	5.388914	7.09E-08	Whole Blood
SH2B3	12	rs653178	6.66	5.33521	9.54E-08	Whole Blood
FAM177B	1	rs17163358	−6.711	5.269907	1.36E-07	Whole Blood
TAF1A	1	rs17163358	−6.711	5.266864	1.39E-07	Whole Blood
BSND	1	rs11591147	−5.173	5.15595	2.52E-07	Whole Blood
UBE2Q1	1	rs4845618	5.284	5.015	5.30E-07	Whole Blood
RP11–422P24.10	1	rs4845618	5.284	−4.984054	6.23E-07	Whole Blood
CDKN2A	9	rs4977574	18.33	−4.94884	7.47E-07	Whole Blood
SRD5A3-AS1	4	rs11945371	−3.16	−4.911178	9.05E-07	Whole Blood
FES	15	rs8039305	5.36	−4.77814	1.77E-06	Whole Blood
RP11-37E23.5	13	rs7328733	−4.91	4.7098	2.48E-06	Whole Blood
MIA3	1	rs17163358	−6.711	4.631167	3.64E-06	Whole Blood
HIC1	17	rs2760740	4.83	−4.62824	3.69E-06	Whole Blood
SREBF1	17	rs16960744	4.57	−4.5888	4.46E-06	Whole Blood
IP6K2	3	rs34759087	−4.52	4.56636	4.96E-06	Whole Blood

### Identification of Differentially Expressed Genes and Functional Enrichment Analyses in the Peripheral Blood of Patients With AMI

**FIGURE 1:**
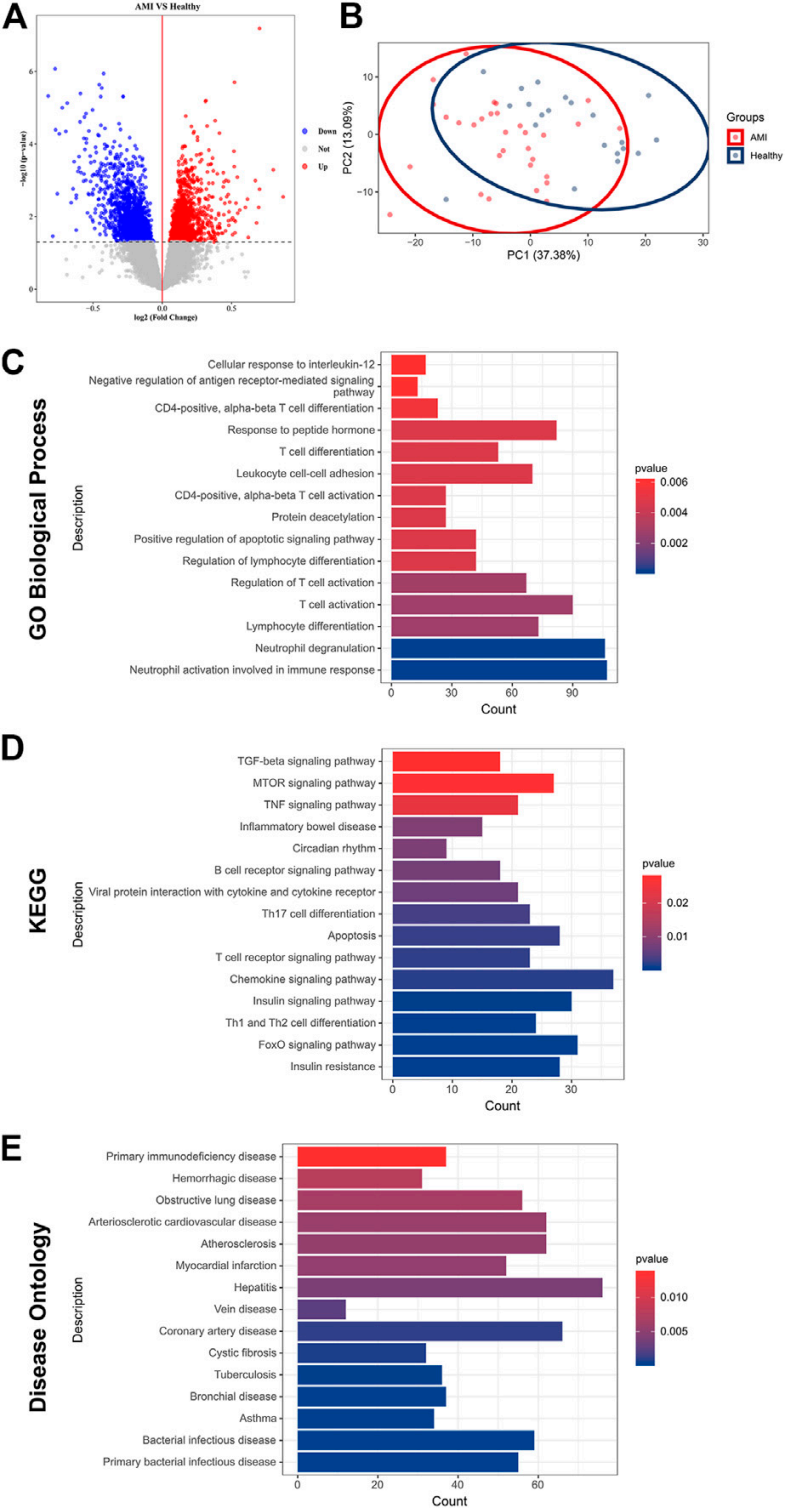
Identification of DEGs and functional enrichment in AMI. **(A)** A volcano plot showing DEGs in AMI samples and healthy controls (*p* < 0.05). The up-regulated genes are marked in red, and the down-regulated genes are marked in blue; **(B)** PCA of DEGs showing good differentiation power; **(C)** GO biological process enrichment analysis of DEGs; **(D)** KEGG enrichment analysis of DEGs; **(E)** DO enrichment analysis of DEGs.

Subsequent differential analysis was performed to identify differentially expressed genes (DEGs) in the peripheral blood samples of AMI patients and healthy controls. A threshold of fold change (FC) > 1 and *p* < 0.05 was set to avoid omission. A total of 3,324 DEGs were identified; of which, 1755 were up-regulated and DEGs were down-regulated (Figure 1A). Principal component analysis (PCA) revealed that these DEGs allowed differentiation between AMI samples and healthy controls (Figure 1B).

To further investigate the pathophysiological functions of these DEGs, Gene Ontology (GO), Kyoto Encyclopedia of Genes and Genomes (KEGG) and (DO) enrichment analyses were performed using clusterProfiler. GO analysis revealed that the DEGs were mainly involved in T cell activation, lymphocyte differentiation and adhesion and immune response (Figure 1C). According to KEGG analysis, the DEGs were involved in various classical signalling pathways, including transforming growth factor-beta (TGF-*β*), mammalian target of rapamycin (mTOR), tumour necrosis factor (TNF), forkhead box O3 (FoxO) and chemokine signalling pathways. In addition, they were also involved in the regulation of T and B cell activity and apoptosis (Figure 1D). Furthermore, DO analysis revealed the enrichment of DEGs in several CVDs, including atherosclerosis, coronary artery disease (CAD) and myocardial infarction (Figure 1E). These results confirmed a high correlation between DEGs and AMI and that DEGs mainly regulated immune cell activity and apoptosis.

### Identification of FRGs Specifically Expressed in the Peripheral Blood of Patients With AMI

**FIGURE 2:**
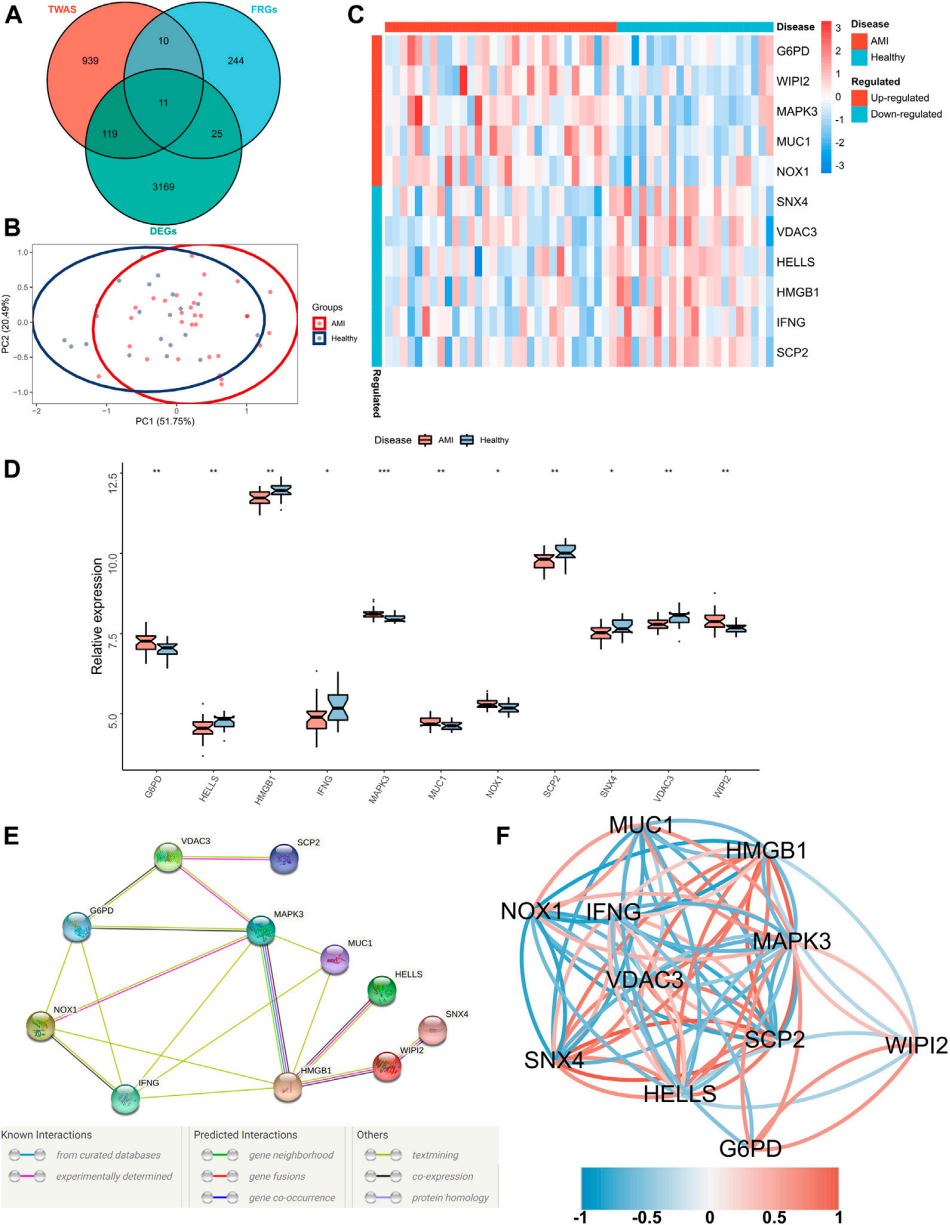
Identification of FRGs specifically expressed in the peripheral blood of patients with AMI. **(A)** A Venn diagram showing the intersection of TWAS results, DEGs and obtained FRGs, wherein 11 shared FRGs were identified; **(B)** PCA of FRGs showing good differentiation power; **(C)** A heatmap showing the transcriptional profiles of FRGs in AMI samples and healthy controls; **(D)** Box plots showing differential expressions of FRGs in AMI samples and healthy controls. Wilcoxon test; *p < 0.05, **p < 0.01, ***p < 0.001; **(E)** PPI network of FRGs; **(F)** Correlation network of FRGs.

Significant gene expression–trait associations were identified using TWAS; therefore, we integrated the results of TWAS and differential analyses and intersected them with the obtained FRGs. Consequently, 11 FRGs were obtained in the peripheral blood samples of patients with AMI (Figure 2A). In addition, PCA revealed that these genes could well differentiate between AMI samples and healthy controls (Figure 2B). Furthermore, a heatmap was created to visualise significant differences in the expression of these genes between AMI samples and healthy controls (Figure 2C). The expression of lymphoid-specific helicase (HELLS), high-mobility group box 1 (HMGB1), interferon-gamma gene (IFNG), sterol carrier protein 2 (SCP2), sorting nexin 4 (SNX4) and voltage-dependent anion channel 3 (VDAC3) was significantly low, whereas that of glucose-6-phosphate dehydrogenase (G6PD), mitogen-activated protein kinase 3 (MAPK3), mucin 1 (MUC1), NADPH oxidase-1 (NOX1) and WD repeat domain phosphoinositide-interacting protein 2 (WIPI2) was significantly high in AMI samples (Figure 2D). A protein–protein interaction (PPI) network of these 11 genes was constructed using the String database, which revealed MAPK3 and HMGB1 as the hub genes (Figure 2E). In addition, the correlation network revealed a significant correlation pair between the 11 genes (Figure 2F).

### Construction and Validation of an Optimal Ferroptosis-Related AMI Prediction Model

**FIGURE 3:**
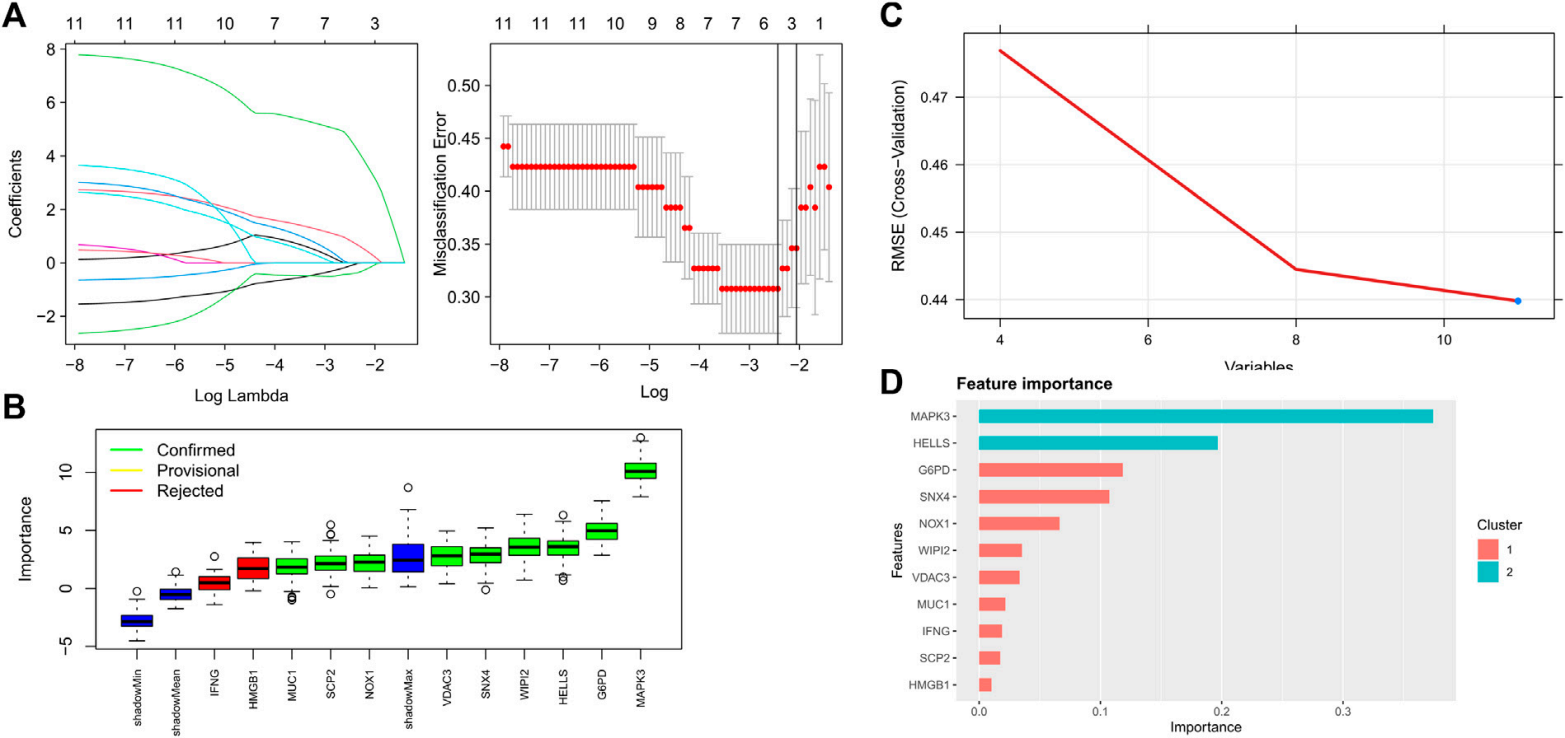
Key FRGs screened using machine learning algorithms **(A)** 4 FRGs obtained using the LASSO algorithm based on the minimum lambda; **(B)** 9 FRGs obtained using the RFB algorithm; **(C)** 11 FRGs obtained using the SVM algorithm; **(D)** 11 FRGs obtained using the XGBoost algorithm.

**FIGURE 4:**
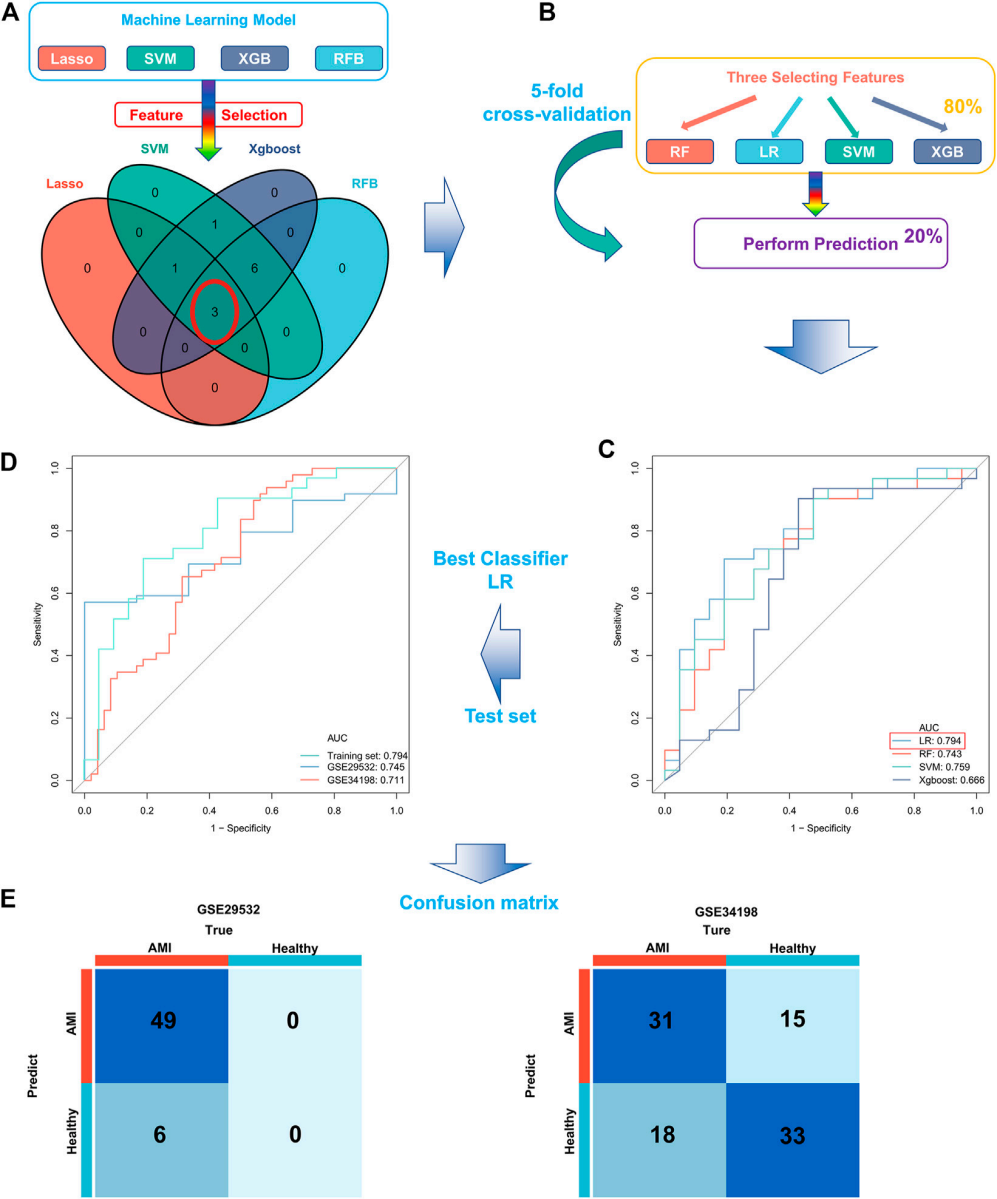
Construction and validation of a ferroptosis-related AMI prediction model. **(A)** Screening of three key FRGs in the GSE48060 dataset using four machine learning algorithms; **(B)** Schematic diagram of training and validation of a stable classifier in the training cohort using four machine learning algorithms based on five-fold cross-validation; **(C)** ROC curves of four predictors based on cross-validation in the training cohort; **(D)** ROC curves for applying the optimal classifier (LR) to two external validation cohorts; **(E)** Confusion matrix of the predictors in two external validation cohorts. Left, GSE29532; right, GSE34198.

Four proven machine learning algorithms (least absolute shrinkage and selection operator [LASSO], random forest and boruta [RFB], support vector machine [SVM] and extreme gradient boosting [XGBoost]) were used to identify key ferroptosis-related features in the training cohort, yielding 4, 9, 11 and 11 genes, respectively (Figure 3). Furthermore, three key genes (MAPK3, WIPI2 and VDAC3) shared by the four algorithms were selected as FRGs to build a prediction model (Figure 4A). Subsequently, we assessed the efficiency of the four supervised machine learning algorithms (logistic regression [LR], random forest [RF], SVM and XGBoost) using receiver operating characteristic (ROC) curves based on five-fold cross-validation (Figure 4B). Classifiers trained on three key FRGs were found to differentiate well among patients with AMI (LR, AUC = 0.794; RF, AUC = 0.743; SVM, AUC = 0.759; Xgboost, AUC = 0.666, Figure 4C). Notably, the LR model exhibited the highest AUC. The performance of the four algorithms was subsequently evaluated in detail, and the results are presented in Table 2. The LR model had the highest Kolmogorov–Smirnov (KS) value, demonstrating a high efficiency in differentiating between AMI samples and healthy controls (KS = 0.519). In addition, the LR model had the best accuracy (accuracy = 0.692). However, because AMI is a severe acute disease, patients with AMI need to be identified more accurately; therefore, recall is equally important. Satisfactorily, the LR model also had the highest recall (recall = 0.75).

**TABLE 2:** Comparison of the diagnostic efficacy of four different machine learning models.

Model	TP	TN	FP	FN	Precision	Recall	F1 score	Accuracy	KS	Error
LR	24	13	7	8	0.774193548	0.75	0.761904762	0.692307692	0.5192012	0.307692308
RF	24	12	7	9	0.774193548	0.727272727	0.75	0.673076923	0.4270353	0.326923077
SVM	24	11	7	10	0.774193548	0.705882353	0.738461538	0.692307692	0.4270353	0.307692308
Xgboost	24	12	7	9	0.774193548	0.727272727	0.75	0.461538462	0.4746544	0.538461538

**FIGURE 5:**
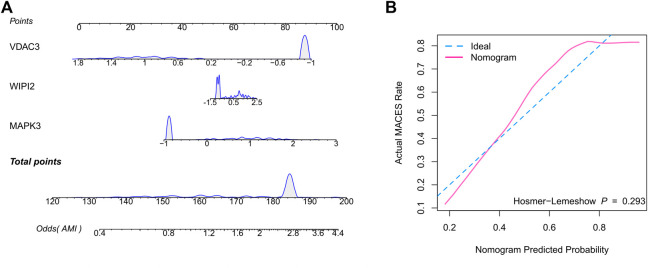
Construction of the nomogram based on the logistic regression model. **(A)** Nomogram specifically quantified the odds ratio of AMI based on 3 ferroptosis characteristics. **(B)** The calibration curves of nomogram.

Therefore, we hypothesized that LR may serve as the best prediction model. The predictive efficiency was validated by applying the LR model to two external cohorts. The ROC curves exhibited satisfactory efficiency of the model with an AUC value of 0.745 in the GSE29532 dataset and 0.711 in the GSE34198 dataset (Figure 4D). In addition, a confusion matrix was used to visualise the efficiency of the classification model (Figure 4E). Notably, the classifier exhibited satisfactory efficiency in the GSE29532 dataset, and all patients with AMI were correctly identified, with only six healthy individuals being misidentified as patients with AMI. However, the small number of healthy controls in this dataset might have created a bias in assessing the efficiency of the classifier. Moreover, in the GSE34198 dataset, the classifier exhibited good efficiency and correctly identified 31 patients with AMI and 33 healthy individuals; however, 18 patients with AMI were incorrectly identified as healthy individuals. Considering the accurate predictive efficacy of the LR model, a nomogram was constructed to estimate the odds ratio of AMI more clearly (Figure 5A). According to the calibration curve and hosmer-lemeshow test (*p* > 0.05), the nomogram was accurate and robust (Figure 5B).

## Discussion

AMI, a common and highly prevalent CAD worldwide, can cause malignant arrhythmias and heart failure, resulting in high mortality and disability (Roger et al., 2012; Mozaffarian et al., 2016). Advancements in thrombolytic and interventional techniques have significantly improved the prognosis of patients with AMI. However, owing to the low specificity and sensitivity of existing myocardial markers, a large proportion of patients fail to receive prompt treatment, resulting in irreversible myocardial damage and the eventual occurrence of heart failure and arrhythmias (Braunwald, 2012; Eapen et al., 2012). Early diagnosis can be effective in improving the prognosis and reducing the mortality of patients with AMI. Therefore, it is essential to identify effective diagnostic biomarkers and develop diagnostic models for AMI.

In this study, we systematically screened for FRGs specifically expressed in the peripheral blood of patients with AMI and build a stable AMI diagnostic model integrating three key ferroptosis-related markers (MAPK3, WIPI2 and VDAC3) using multiple machine learning algorithms. In addition, the predictive efficiency of the diagnostic model was evaluated in two external cohorts.

To develop a robust diagnostic model for AMI, TWAS was initially performed using large-scale GWAS data on AMI to identify feature genes in the peripheral blood of patients with AMI. Subsequent differential analysis of mRNA expression profiles identified 3,324 DEGs. Furthermore, functional annotation identified the primary involvement of DEGs in immune response and multiple classical signalling regulatory pathways, including TGF-*β*, mTOR, TNF, FoxO and chemokine signalling pathways, suggesting that the main biological processes involved in the progression of AMI are inflammatory and immune responses. Moreover, further enrichment analyses revealed an important role of DEGs in various CVDs.

Ferroptosis plays a positive regulatory role on immune function in an inflammatory environment (Wang et al., 2019; Kapralov et al., 2020). Recent studies have reported promising applications of ferroptosis in the prevention of CVDs (Gao et al., 2015; Stockwell et al., 2017; Fang et al., 2019). Therefore, we considered FRGs as potential biomarkers and integrated the results of TWAS and DEG and FRG expression data to screen for FRGs in the peripheral blood of patients with AMI. For clinical convenience and cost reduction, we used four machine learning algorithms (LASSO, RFB, SVM and XGBoost) and eventually proposed a diagnostic model comprising three FRGs (MAPK3, WIPI2, and VDAC3). MAPK3 plays a key role in cell differentiation, cell proliferation, stress response and apoptosis when the heart receives pathophysiological stimuli (Purcell et al., 2007; Gutkind and Offermanns, 2009; Lorenz et al., 2009). It has been suggested that MAPK3 induces cardiac hypertrophy in response to pathological injury in the heart (Purcell et al., 2007; Lorenz et al., 2009; Kehat and Molkentin, 2010). Furthermore, another study has demonstrated that MAPK3 downregulation leads to apoptosis in cardiac myocytes (Liu et al., 2018). These studies suggest that MAPK3 expression increases in the presence of myocardial ischaemia, exerting a protective effect to inhibit apoptosis and hence maintaining normal ejection function through compensatory hypertrophy (Lorenz et al., 2009; Gartz et al., 2018; Deng et al., 2021). However, over-activated MAPK3 can lead to dilated cardiomyopathy and heart failure (Huby et al., 2016). Our study showed increased MAPK3 expression in patients with AMI, thus providing a basis for early diagnosis. However, considering the adverse impact of MAPK3, new molecular therapeutic strategies should also be developed. WIPI2 is a key protein that promotes the growth and elongation of autophagosomes and mainly regulates autophagy in cells. Therefore, degradation of WIPI2 can effectively inhibit autophagy (Wan et al., 2018; Lu et al., 2019; Wan and Liu, 2019). Previous studies have suggested that activating autophagy plays a cardioprotective role in cases of myocardial ischaemia. However, sustained autophagy can also lead to heart failure (Nishida et al., 2009; Sciarretta et al., 2018). The functional role of autophagy in cardiac ischaemia/reperfusion is complex, and targeting autophagy has been suggested as a potential therapy for myocardial injury (Delbridge et al., 2017). To the best of our knowledge, the present study is the first to report that WIPI2 is highly expressed in AMI, thus providing novel insights into the role of autophagy and pharmacological intervention in myocardial ischaemia. Furthermore, VDAC3 is mainly found in the mitochondrial outer membrane and is responsible for transporting low-molecular-weight metabolites. Therefore, mitochondrial dysfunction due to VDAC3 alterations can lead to apoptosis and several diseases (Reina et al., 2016; Karachitos et al., 2017; Chin et al., 2018). Consistent with our study, several studies have reported a decrease in VDAC3 expression after the treatment of cerebral ischaemia (Yao et al., 2018), suggesting that VDAC3 prevents mitochondrial damage and improves tissue function after ischaemia. Therefore, VDAC3 may also be a potential therapeutic target for AMI and requires further investigation.

Machine learning has a wide range of applications in biomedicine and exhibits excellent efficiency in clinical diagnosis and optimal treatment (Rajkomar et al., 2019; Do and Le, 2020; Goecks et al., 2020; Le et al., 2021). In this study, the predictive power of four machine learning classifiers (LR, RF, SVM and XGBoost) was evaluated to build a stable LR-based AMI prediction model, which showed excellent predictive power in the training cohort (AUC = 0.794, accuracy = 0.692). Furthermore, the prediction model exhibited good efficiency in two external validation cohorts (AUC = 0.745 and 0.711), providing new insights into early and rapid diagnosis of AMI. Chen et al. also developed a RF diagnostic model of AMI, the AUC value is 0.855 (train set) and 0.731 (test set) (Yifan et al., 2021). Fang et al. developed a SVM diagnostic model of AMI, the AUC value is 0.860 (train set) and 0.921 (test set) (Fang et al., 2020). Compared with two previous studies, our model showed satisfactory accuracy in both two external validation data, suggested that our model was more robust and universal. However, limited by the small sample size, the prediction model did not have satisfactory accuracy and recall in the external validation cohort, which led to misdiagnosis and missed diagnoses. Therefore, larger AMI cohorts can better train a diagnostic model to improve the prediction accuracy.

## Materials and Methods

### Data Acquisition

The mRNA expression profiles of patients with AMI were obtained from three GEO databases, namely, GSE48060, GSE29532 and GSE34198. Samples for all three datasets were collected from the peripheral blood of patients with AMI. The GSE48060 dataset (Suresh et al., 2014), which was generated using platform GPL570, was used as the training cohort for variable screening and model training. The GSE29532 and GSE34198 datasets, from platforms GPL5175 and GPL6102, respectively (Silbiger et al., 2013), served as external validation datasets of the model to avoid batch effects. All datasets were log2 normalised.

In addition, large-scale GWAS data on CAD were obtained from CARDIoGRAMplusC4D, including 60,801 clinical cases and 123,504 controls from 48 GWAS meta-analyses of CAD (Nikpay et al., 2015; Luo et al., 2019). The selected cases that belonged to the MI subgroup constituted approximately 70% of the total number of cases. Refer to the original study (Nikpay et al., 2015) for specific information on the dataset.

### TWAS Analysis

In this study, the FUSION software was used for performing TWAS on patients with AMI (Gusev et al., 2016). Briefly, tissue-specific gene expression was obtained based on GWAS data and gene expression data using whole-blood gene expression data from the GTEx consortium as reference weights. Subsequently, the imputed gene expression was correlated with traits to evaluate the association of each gene with a given disease. Furthermore, potential AMI-related genes were screened, with a threshold of FDR<0.05.

### Identification of DEGs and Functional Enrichment Analyses

In this study, differential expression analysis was performed using the R package “limma”. To avoid omission, DEGs were screened at a threshold of *p* < 0.05, and the efficiency of DEGs was evaluated *via* PCA. Subsequently, functional enrichment analyses of DEGs, including GO, KEGG and DO, were performed using the R package “clusterProfiler” (Yu et al., 2012), pathways with FDR <0.05 were considered significant.

### Identification of Differential FRGs

FRGs were obtained from the FerrDb database (Zhou and Bao, 2020) (http://zhounan.org/ferrdb) and previous studies (Stockwell et al., 2017; Hassannia et al., 2019; Doll et al., 2019; Bersuker et al., 2019). Supplementary Table S1 enlists the FRGs included in this study. The intersection genes of TWAS, DEGs and FRGs were considered FRGs specifically expressed in the peripheral blood of patients with AMI and were used for further analysis. Subsequently, a PPI network of the FRGs was constructed using the String database (http://string-db.org/) (Szklarczyk et al., 2015). The correlation among FRGs was assessed using Pearson correlation coefficient, and Cytoscape (version 3.7.1) was used to visualise the correlation network.

### Robust Predictive Model Built Using Multiple Machine Learning Methods

The R packages glmnet, rms, e1071, randomForest, Boruta and XGBoost were used to build a machine learning model (Sauerbrei et al., 2007; Kim, 2014; Li et al., 2019; Yperman et al., 2020). First, LASSO regression (nfold = 5, type. measure = “class”), SVM (number = 20), RFB (doTrace = 2, ntree = 1,000, maxRuns = 100), and XGBoost (max_depth = 2, eta = 1, silent = 1, nround = 25) analyses were performed on the entire dataset to screen for key FRGs. Consequently, the intersection genes obtained *via* analyses were considered the key FRGs associated with AMI and were used to further construct and train a prediction model. Subsequently, the efficiency of the prediction model was assessed *via* five-fold cross-validation in the dataset. Specifically, the GSE48060 dataset was divided into five equal parts, wherein 4/5 of the training data was used to train the prediction model. Subsequently, the trained model was applied to the remaining 1/5 of the training data for prediction. We integrated prediction results from the five iterations and evaluated the efficiency of the classifier by plotting ROC curves and using a confusion matrix. Eventually, we considered LR as an optimal classifier to build a prediction model for AMI and applied it to two external validation cohorts to assess the generalisation ability.

## Data Availability

Publicly available datasets were analyzed in this study. This data can be found here: The mRNA expression profiles of patients with AMI were obtained from three GEO databases (https://www.ncbi.nlm.nih.gov/gds/), namely, GSE48060, GSE29532, and GSE34198.In addition, large-scale GWAS data on CAD were obtained from CARDIoGRAMplusC4D, including 60,801 clinical cases and 123,504 controls from 48 GWAS meta-analyses of CAD.
